# Rapid increase in growth and productivity can aid invasions by a non-native tree

**DOI:** 10.1093/aobpla/plw048

**Published:** 2016-08-02

**Authors:** Rafael Dudeque Zenni, Wanderson Lacerda da Cunha, Guilherme Sena

**Affiliations:** Department of Ecology, University of Brasília, Campus Universitário Darcy Ribeiro, Brasília CEP 70910-900, Brazil

**Keywords:** Biological invasions, contemporary evolution, exotic species, growth-defence trade-offs, invasion biology, invasiveness, *Pinus taeda*, range expansion, tree invasions

## Abstract

Research on biological invasions has produced detailed theories describing range expansions of introduced populations. However, current knowledge of evolutionary factors associated with invasive range expansions, especially those related to rapid evolution of long-lived organisms, is still rudimentary. Here, we used a system of six 40-year-old invasive pine populations that originated from replicated introduction events to study evolution in productivity, growth, and chemical defence traits. We tested the hypotheses that invasive populations were undergoing rapid phenotypic change as populations spread, that populations exhibit trade-offs between evolution in growth and chemical defences, and that rates of rapid evolution in plant growth and productivity effect rates of invasion. Although all invasions started from replicated pools of genetic material and equal propagule pressure, we found divergence in mean values for the six invasive populations in the six traits measured. Not only were there between-population variations but also invasive populations were also rapidly changing along each invasive population expansion. Two populations displayed greater leaf areas (LAs) and smaller specific LAs (SLAs) during range expansion. Four populations had faster growth rates at the leading edge of the invasion front in comparison with plants at the rear edge. In terms of total plant defences, non-volatile resin increased in plants along one invasion gradient and decreased in a second, total needle phenolics increased in plants along one invasion gradient and total wood phenolics increased in plants along the one invasion gradient and decreased in a second. We found no trade-offs between investments in growth and chemical defence. Also, faster rates of change in growth rate and LA were positively associated with greater dispersal distances of invasive populations, suggesting rapid evolution may increase invasiveness. Understanding the roles of both natural and human-mediated ecological and evolutionary processes in population-level dynamics is key to understanding the ability of non-native species to invade.

## Abstract (precis)

Biological invasions are currently an important cause of environmental degradation, but they also offer important insights on how species respond to changes in the environment. Invasive species affect native ecosystems, but they are also affected by them. Our new study evaluated how invasive populations of a non-native pine responded to climate and other environmental factors. Our results show that many plants at the forefront of six different invasions are growing faster and being more productive than the plants originally planted 40 years ago. Taken together, the capacity for adaptation to different conditions and evolution of increased growth rates make *Pinus taeda* an invasive species that requires management and control before its spread reaches large areas.

## Introduction

Biological invasions are a leading cause of environmental degradation, are a main focus of concern for conservation practitioners and provide important insights on species responses to climate change (Pyšek *et al.* 2012; Caplat *et al.* 2013; Moran and Alexander 2014; Kuebbing and Simberloff 2015). Invasive organisms alter ecosystem properties and community dynamics, impacting native species and ecosystem functioning (Wardle *et al.* 2011; Yelenik and D'Antonio 2013). Driving the spread and impact of non-native populations are dynamic ecological and evolutionary processes acting at levels ranging from genes to global scale (Zenni 2014; Zenni *et al.* 2014; Zenni and Hoban 2015b). Several decades of ecological research have produced detailed frameworks and theories describing range expansions of introduced populations (e.g. Blackburn *et al.* 2011; Gurevitch *et al.* 2011). More recently, researchers started to disentangle the evolutionary mechanisms driving spread of non-native populations and to incorporate them into invasion theory (Parker *et al.* 2003; Prentis *et al.* 2008; Sargent and Lodge 2014). However, the role of contemporary evolution in invasive range expansions is still poorly understood (Colautti and Barrett 2013; Zenni *et al.* 2014), especially for long-lived organisms such as trees.

Climate has been hypothesized to be a major selection force on adaptation of invasive plant populations, changing plant phenology and size (Colautti and Barrett 2013; Colomer-Ventura *et al.* 2015). However, evolution can also occur in growth rates and defence traits (Buswell *et al.* 2011). Investments in defence against herbivory is costly for plants, and resource allocation for chemical defences can limit plant growth or reproduction (Coley *et al.* 1985; Mithöfer and Boland 2012). The well-established relationship of constant trade-offs among investment in growth, reproduction and defence traits has led to the formulation of the ‘evolution of increased competitive ability’ hypothesis, which states that introduced plants are liberated from natural enemies and, thus, can allocate towards growth and reproduction resources previously required for defence (Blossey and Nötzold 1995). By increasing their resource investment in growth and reproduction, non-native populations may become abundant and widespread (Keane and Crawley 2002). Indeed, many invasives, including pines, escape from natural enemies when introduced to a new range (Liu and Stiling 2006). In their native ranges, several species of *Pinus* exhibit trade-offs between growth rates and chemical defences; slow-growing species and populations invest more in constitutive defences (Moreira *et al.* 2014). For non-native populations, the absence of significant herbivory pressure may reduce plant resource allocation towards defence, possibly increasing fitness of non-native populations. Consequently, invasive populations may evolve increased growth rates and reproduction during the invasion process.

Besides local adaptation, genetic drift and phenotypic plasticity, the human-mediated introduction of pre-selected and adapted genetic lineages may also produce fit organisms in non-native ranges and benefit range expansions (Zenni *et al.* 2014). The introduction of highly variable groups of individuals may produce the same effect (Forsman *et al.* 2012; Zenni and Simberloff 2013; Forsman and Wennersten 2015). Further, the co-introduction of previously allopatric populations can lead to genetic recombination and novelty, possibly increasing levels of heterozygosity and polymorphism, which could trigger invasions that may not occur based on the original genotypes alone. However, the importance of admixture during invasions has not received much support from the literature (Rius and Darling 2014). While local adaptation, drift and phenotypic plasticity act during the naturalization stage and are part of all natural systems, the latter two mechanisms (introduction of pre-selected and adapted genetic lineages, and introduction of highly variable groups of individuals) act prior and during the introduction stage and may be exclusive to human mediated biological invasions (as opposed to natural range expansions, even those owned to human-mediated climate change). The processes acting at each stage of the invasion process can be different, and most studies have lumped together species at different stages of the introduction–naturalization–invasion continuum, or have looked only at processes occurring at the invasion stage (Blackburn *et al.* 2011; Richardson and Pyšek 2012; Moodley *et al.* 2013). Thus, understanding the roles of both natural and human-mediated agents prior to and during naturalization is key to understanding the invasion process. For this, researchers need to know the history of the introduction, including number and diversity of propagule sources, and follow the fate of the non-native populations during naturalization and invasion (Burton *et al.* 2010; Donaldson *et al.* 2014).

For animals, several studies have shown association between evolutionary dynamics and invasive range expansions. Invasive cane toads (*Rhinella marina)*, for instance, evolved longer legs in the 70 years since introduction in Australia, increasing 5-fold the annual rate of expansion of the toad invasion front (Phillips *et al.* 2006). Also, for invasive European starlings (*Sturnus vulgaris*) in South Africa, unfavourable environmental conditions enhanced dispersal, which preserves genetic diversity during range expansion and reduces potentially detrimental founder effects (Berthouly-Salazar *et al.* 2013). For lizards (*Podarcis muralis*) invading in Germany, a study found a trend for an increase in genetic differentiation and a decrease in genetic diversity from the invasion centre to the expanding range edge, suggesting genetic drift as a major factor in the structuring of populations (Schulte *et al.* 2013). For plants, virtually all examples of rapid evolution during invasive range expansions come from short-lived species. For example, in North America, local adaptation of purple loosestrife (*Lythrum salicaria*) in response to climate allowed populations to spread northward (Colautti and Barrett 2013). Further, the great majority of studies on evolution of invasive plants have compared invasive and native populations, or have compared distinct invasive populations. Very few studies have studied populations along invasion gradients.

**Figure 1.**
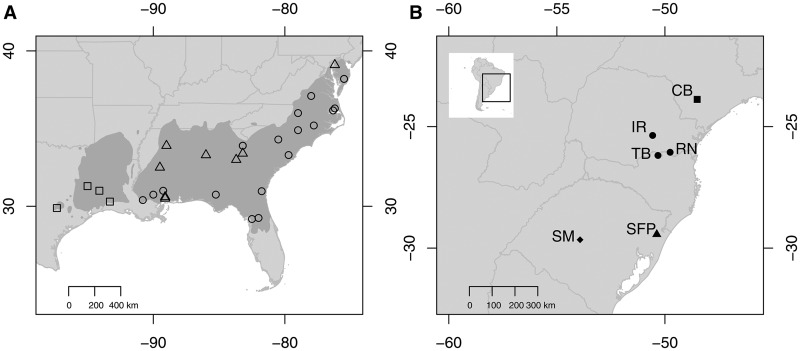
Origin of the *Pinus taeda* seed sources in the USA and location of common gardens and invasive populations in Brazil. (A) Light grey represents the continental USA, dark grey represents *P. taeda* native range, and dots are the location of the 32 seed sources planted in the six common gardens. Open symbols represent the three genetic provenances. (B) Location of the six common gardens in Brazil. Solid symbols (circles, triangle, diamond and square) represent different climates. CB, Capão Bonito; IR, Irati; RN, Rio Negro; SFP, São Francisco de Paula; SM, Santa Maria; TB, Três Barras.

In order to evaluate the role of human-mediated and natural evolutionary processes (natural selection and genetic drift) in the invasion success of non-native tree species, we designed a study using six fully replicated common garden experiments in southern Brazil where *Pinus taeda* L. (loblolly pine) was introduced at the same time (1973 and 1975), in the same numbers, from the same seed sources, and has formed invasive populations expanding outward from the plantations (i.e. invasive range expansions). *Pinus taeda* is a long-lived forest tree that has multi-generational populations, reproduces early (5 years) and yearly, and is wind-dispersed with viable seed dispersal distances of less than 20 m (Zenni *et al.* 2014). The common garden experiments were originally planted to serve as forestry provenance trials for silvicultural purposes (Shimizu and Higa 1981). The experiments encompass a north-south transect covering about 850 km or 6° of latitude (Fig. 1). Previous work on the system showed provenance-by-environment interactions where genetic lineages of *P. taeda* exhibited differential naturalization success depending on the climate of the location into which they were introduced (Zenni *et al.* 2014). Furthermore, 25 genes were undergoing significant shifts in allele frequencies along the invasion gradients (Zenni and Hoban 2015b). Although the genes evolving were mostly population specific, many were associated to important plant functions, such as phloem sugar transport, nitrate uptake, and pollen tube growth (Zenni and Hoban 2015b). Taken together, both studies make it evident that *P. taeda* is undergoing rapid evolution in all six invasive populations (Zenni *et al.* 2014).

Here, we aimed to identify phenotypic changes undergone by the six populations of *P. taeda* in order to see how rapid evolution and genotype-by-environment interactions altered these invasive populations in growth, productivity, and chemical defence traits. We tested the following hypotheses: (i) the observed genotypic changes resulted in phenotypic changes for growth, productivity, and chemical defence traits; (ii) patterns of phenotypic changes match patterns of genetic change across populations; (iii) populations exhibit trade-offs between evolution in growth and chemical defences; and (iv) rates of rapid evolution in plant growth and productivity effect rates of invasion.

## Methods

### Study system

In 1973 and 1975, six common garden experiments for *P. taeda* were established in Brazil at the Santa Maria Experimental Farm (53.92°W 29.66°S), São Francisco de Paula National Forest (50.38°W 29.43°S), Três Barras National Forest (50.32°W 26.19°S), Rio Negro Experimental Station (49.76°W 26.05°S), Irati National Forest (50.57°W 25.36°S), and Capão Bonito National Forest (48.51°W 23.88°S). The six locations represent four climates and two of these four climates (Santa Maria and Capão Bonito) are different from the climate in *P. taeda* native range. Annual precipitations vary from 1212 mm to 2068 mm, whereas mean annual temperatures vary from 15.2 °C to 19.1 °C. Both precipitation and temperature patterns exhibit clinal variations from north to south. Climate types were determined using multivariate clustering analyses with data from Worldclim (see Zenni *et al.* 2014 for details). Furthermore, Irati, Três Barras, Rio Negro and São Francisco de Paula original ecosystem are forest (Araucaria ombrophilous forest), Capão Bonito is a neotropical savanna (cerrado), and Santa Maria is a grassland ecosystem. Previous work showed no effect of original ecosystem type on the genetic changes observed for *P. taeda* (Zenni *et al.* 2014; Zenni and Hoban 2015b).

The common gardens were planted with 29 or 32 seed sources of which 20 were present in all gardens (Zenni *et al.* 2014). Each seed source corresponded to a seed lot collected from between 5 and 10 trees in natural stands in the species’ native range (Fig. 1) [**see Supporting Information**]. In each Brazilian common garden, seed sources were planted in randomized blocks with four repetitions—a total of 144 trees from each seed source were planted in each common garden in four randomly placed squares of 6×6 trees (Shimizu and Higa 1981). Over the years, each common garden and its surroundings received circumstantial and haphazard management (e.g. some of the common gardens were thinned and plants growing on fire breaks and along roads were cut). The invasive populations themselves received only minor and dispersed management interventions. There were also high mortality rates for some seed sources in the common gardens probably owed to the high density of the plantations. In November and December 2014, all seed sources were still represented by at least 10 trees at any given garden, but the mean number of trees per seed source per site was usually higher. Previous work showed the seed sources form three native range genetic provenances (a provenance is an environmentally explicit genetic delineation of seed sources, and may be defined by a genetic cluster) and that the spatial distribution of these provenances across *P. taeda* native and invasive ranges correlates with temperature and precipitation patterns (Zenni *et al.* 2014). The common gardens are considered parallel replicated introduction pools resulting in identical propagule pressures and residence times for these six locations (Zenni *et al.* 2014; Zenni and Hoban 2015b).

Since introduction, the common gardens have produced invasive populations expanding between ca. 100 and 450 m from the common garden. By sampling plants at different distances from each of the common gardens, from the border of the plantations to the leading edge of the invasion fronts, we were able to track changes in genotype and phenotype frequencies in these six naturalized populations over multiple generations encompassing 40 years of population growth. This approach already showed that the six naturalized populations are undergoing contemporary evolution and significant genetic changes are occurring along the invasive range expansions. At the provenance level, genetic changes were associated with climate (Zenni *et al.* 2014), whereas at the gene level, genetic changes were mostly population specific (Zenni and Hoban 2015b).

### Data collection and trait measurements

The experiment design, description of sample and data collection, and specifications of the laboratory and genotyping methods used for the genetic work have been detailed previously (Zenni *et al.* 2014; Zenni and Hoban 2015b). Briefly, 50 plants were haphazardly sampled from each of six invasive *P. taeda* populations (a total of 300 plants) and genotyped for 94 single nucleotide polymorphisms (SNPs) using Fluidigm® SNPtype Assays. Plants sampled were at least 1.3 m tall and had between 3 and 34 years of age. For this study, the original plantings were not included because traits from trees growing at high-density monocultures may not be comparable with trees naturally growing in heterogeneous habitats. All the SNPs chosen were a subset of the ones used by Eckert *et al.* (2010) and were located in functional genes. Complete genotype data is available at Dryad data repository (Zenni and Hoban 2015*a*, *b*). The distances between the plants and the edge of the common gardens were measured using the function “Hub distance” in the package MMQGIS for QGIS (Quantum GIS Development Team 2015), and distances were normalized for each population (divided by the maximum distance of spread) to account for variations in spread rates across sites (D_NORM_). For the phenotypic part of the study, fieldwork was carried out in November 2014 and January 2015. To collect phenotypic data, we visited the same trees previously sampled for genotyping in all six invasive populations. However, between 2012 (original sampling) and 2014 (sampling for this study), some trees died owing to idiosyncratic factors (i.e. tree falls and establishment of firebreaks). The number of surviving individuals was 47 for Capão Bonito, 48 for Irati, 49 for Rio Negro, 49 for Três Barras, 40 for São Francisco de Paula and 50 for Santa Maria, for a total of 283 plants. We used the same individuals from previous studies and did not include new plants because the sampled plants had already been genotyped.

For each plant, we collected an increment core at the base of the tree (∼ 20 cm from the ground) using a 30 cm increment borer (Haglöf Sweden), a handful of fully developed and healthy needles from the tip of the lowest branch, and a ∼15 cm segment of wood from the tip of the lowest branch. We also measured circumference at breast height (CBH; 1.30 m), tree total height, and bark thickness. All fresh wood and needle samples collected were stored in a freezer (−20 °C) immediately upon arrival at the laboratory. Increment cores were air dried, mounted in wooden supports and sandpapered to improve measurement accuracy. Lengths of growth rings were measured using a digital calliper to determine annual growth, and growth rings were counted to estimate the number of years contained in the extracted core (plant age). For all the analyses, we used the plants’ mean annual growth (MAG). We measured the area of 20 fully developed and healthy needles using a table scanner (Epson Perfection™ V700 Photo) and Digimizer v. 4.3.0 (MedCalc Software), and divided the total area by the number of needles to calculate mean leaf area (LA) of each plant. To determine specific LA (SLA), we dried the previously measured needles at 70 °C for 72 h and weighted them (leaf mass; LM) immediately after removing from the drying oven (SLA = LA×LM^-1^). To estimate inductive and constitutive plant defences in our studied plants, we determined non-volatile wood resin content, needle total phenolic content, and wood total phenolic content. Resin and phenolic extraction and determination followed the procedure described by Moreira *et al.* (2012). Briefly, resin was extracted from 15 cm long × ∼ 0.5 cm wide pieces of wood by mixing them with a solvent (hexane) in an ultrasonic bath at 45 °C for 20 min followed by a 24 h rest (performed twice to maximize extraction outputs). The solution was filtered in paper filters and left to dry. The residual content was weighted to the nearest 0.0001 g, and divided by the weight of the wood dry mass (oven dried at 80 °C for 24 h) to be expressed as mg g^−1^. Phenolic content was determined colorimetrically by the Folin–Ciocalteu method in a Bel Photonics 2000UV spectrophotometer (Bel Photonics do Brasil Ltda., Osasco, SP, Brazil) at 760 nm, using tannic acid as a standard (Moreira *et al.* 2014). Needles were oven-dried for 72 h at 60 °C, ground in a Wiley mill, and sieved in a 5 mm mesh. For each sample, we used 100 mg of ground needles. The ground samples were transferred to centrifuge tubes, mixed with 5 ml of 70 % acetone and left in the refrigerator at 4 °C for 1 h. The solutions were centrifuged for 10 min at 7800 RPM and 0.3 ml of supernatant was removed and mixed with deionized water to make 1 ml of solution. To this solution, we added 0.1 N NaOH in Na_2_CO_3_ and the Folin–Ciocalteu reagent. After 2 h in the refrigerator, we took the spectrophotometer readings.

### Statistical analyses

First, we performed six analyses of variance with Bonferroni correction of *P* values to test for differences in mean trait values of LA, SLA, MAG, non-volatile resin, and total needle and wood phenolic contents among the six locations. Second, we built generalized linear models with Gamma probability distribution and the inverse link function to test the effects of climate (mean annual temperature and annual precipitation) on each of the traits measured. Climate data were obtained from the Worldclim database (Hijmans *et al.* 2005).

Next, we used linear mixed-effect models to test how LA, SLA, MAG, non-volatile resin, and total needle and wood phenolic contents varied as a factor of distance from the introduction point (D_NORM_) and/or were genetically controlled at each location. The genetic clustering results (provenances) from Zenni *et al.* (2014) were used as the genetic makeup of individual plants. The dispersal model assumed for this study is that spread occurs as new generations establish farther from the point of introduction with some degree of back dispersal from the invasion front to the rear edge. Thus, the studied populations represent six unique invasions set out by six fully replicated introduction events. Distance of the invasive plant to the common garden and genetic clustering data were considered fixed effects, whereas plant age was added as a random effect (this was done to remove the effect of plant age from the model). For these analyses, we tested traits separately. For each trait, we built four models: a full model including distance, provenance and age; a distance model including distance as fixed and age as random factor; a provenance model including provenance as fixed and age as random factor; and a null model including only an intercept and age as random factor. We compared the four models for each trait using a likelihood ratio test and models were considered significant if statistically different from the null model at *α* = 0.05. Coefficients of determination were calculated using likelihood-ratio based pseudo-*r*^2^ and represent the variance explained by fixed effects (Nakagawa and Schielzeth 2013). These analyses were done in R 3.2 using the packages “lme4” v. 1.1-7 for mixed-effect models, and “MuMIn” v. 1.14.0 for pseudo-*r*^2^.

To test the association between mean annual growth rates and chemical defences, we built three linear models for each naturalized population using non-volatile wood resin content, needle total phenolic content or wood total phenolic content as a dependent variable and MAG as an independent variable.

We also used the slopes of the linear mixed-effect models to test if rate of change in the measured traits resulted in increased invasive potential. For this, we built linear models using the mixed-model slopes for each trait at each location as the independent variable and maximum distance of spread at each location as dependent variable. The slope coefficients are the mean slope for each trait of all plants in the model.

Finally, we performed a genome-wide association analysis to identify genes associated with the measured phenotypes. We tested each trait separately and assumed a codominant genetic model. The 94 unlinked SNPs were added as factors to the models and a Bonferroni correction was done to counteract the multiple comparisons problem. The analysis was done in R 3.2 using the package “SNPassoc” v. 1.9-2. SNPs identified as having associations with traits were further investigated for specific functions in the Dendrome database (http://dendrome.ucdavis.edu/DiversiTree/) and in Genbank.

## Results

**Figure 2.**
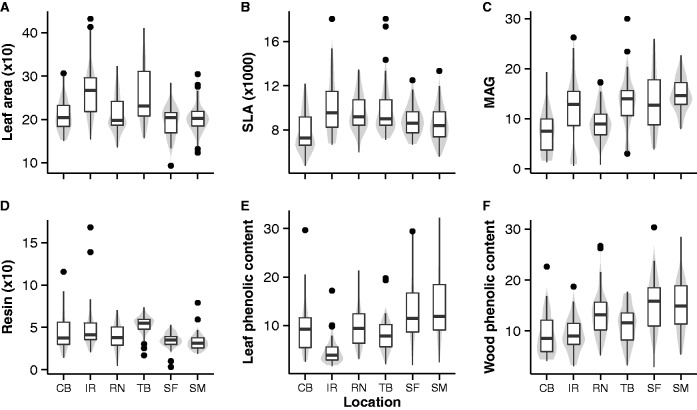
Trait variations among the studied *Pinus taeda* invasive populations. Violin plots and boxplots of (A) leaf area (mm^2^), (B) specific leaf area (SLA, mm^2^/g), (C) mean annual growth (MAG, mm), (D) non-volatile wood resin content (mg/g), (E) total needle phenolic content (mg/g) and (F) total wood phenolic content (mg/g) for each invasive population (CB, Capão Bonito; IR, Irati; RN, Rio Negro; SF, São Francisco de Paula; SM, Santa Maria; TB, Três Barras). Violins (grey areas) are kernel probability densities of the data at different values. Boxplots are median (bold black line), quartiles (white rectangles), standard deviation (black lines) and possible outliers (dots).

**Figure 3.**
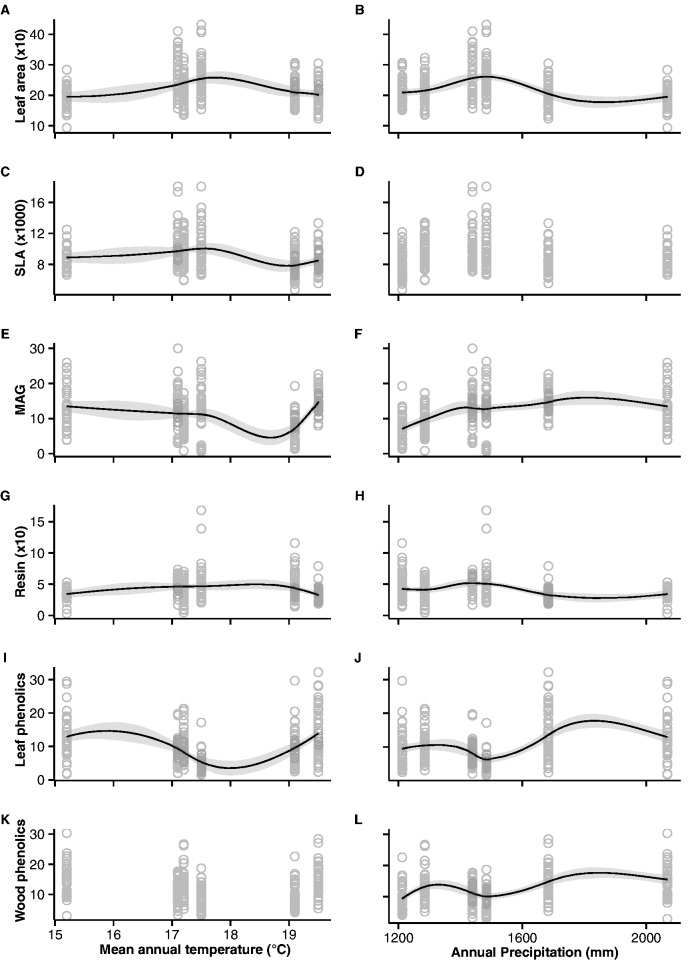
Relationship between leaf area (mm^2^; A and B), specific leaf area (SLA, mm^2^/g; C and D), mean annual growth (MAG, mm/year; E and F), resin (mg/g; G and H), needle phenolic content (mg/g; I and J) and wood phenolic content (mg/g; K and L) and climate (mean annual temperature and annual precipitation) of the six studied locations. Solid lines represent local polynomial regression fitting and grey shades are 95% confidence intervals. Panels D and K show no relationship between variables.

Although all invasions started from replicated pools of genetic material and equal propagule pressure (Zenni *et al.* 2014), we found divergence in mean trait values in the six invasive populations for the six traits measured (Fig. 2 and Table 1): LA (F_5,273_ = 16.69, *P* < 0.001), SLA (F_5,273_ = 7.976, *P* < 0.001), mean annual growth (MAG; F_5,276_ = 15.56, *P* < 0.001), non-volatile resin content (F_5,273_ = 9.945, *P* < 0.001), needle total phenolic content (F_5,273_ = 19.36, *P* < 0.001) and wood total phenolic content (F_5,264_ = 16.63, *P* < 0.001). Climate (mean annual temperature and annual precipitation) explained divergence in LA (*P* < 0.02), SLA (F_3,264_ = 12.1, *P* < 0.001 for MAT), MAG (F_3,264_ = 22.5, *P* < 0.05), resin content (*P* < 0.02), needles phenolic content (*P* < 0.001) and wood phenolic content (*P* < 0.001 for AP) (Table 2 and Fig. 3). While LA and SLA were smaller in hotter and wetter locations, MAG was higher in locations with higher annual precipitations.

**Table 1.** Mean trait values and standard deviation for each invasive population. LA, leaf area; SLA, specific leaf area; MAG, mean annual growth.

	Capão Bonito	Irati	Rio Negro	Três Barras	São Francisco	Santa Maria
LA	210.96±38.64	264.82±61.45	211.45±44.06	257.65±63.79	191.42±39	206.39±40.73
SLA	7904.98±1812.52	10085.93±2657.68	9572.39±1728.83	9849.64±2351.04	9156.25±1682.43	8535.52±1726.33
MAG	7.43±4.18	12.05±6.92	9.2±3.45	13.23±5.39	13.48±6.08	15.27±4.07
Wood resin	43.4±21.79	49.09±26.59	37.93±16.31	52.57±11.12	33.78±10.14	33.16±10.82
Needle phenolics	9.47±5.38	4.75±2.8	10.06±4.56	8.05±3.55	13.07±6.48	13.78±7.07
Wood phenolics	9.2±4.06	9.25±3.44	13.55±4.68	11.33±3.87	15.49±5.52	15±5.33

**Table 2.** Summary results of generalized linear models (Gamma family distribution) for the relationship between plant traits and climate. Null deviance shows how well the response variable is predicted by a model that includes only the intercept, whereas residual deviance shows how well the response variable is predicted by the alternative model. SLA, specific leaf area; MAG, mean annual growth; MAT, mean annual temperature; AP, annual precipitation.

Model	Variable	*t*	*P*	df_Null_	df_Residual_	Deviance_Null_	Deviance_Residual_
Leaf area	MAT	2.40	0.017	267	265	15.282	14.614
	AP	3.18	0.002				
SLA	MAT	3.72	0.000	267	265	13.692	12.936
	AP	1.86	0.065				
MAG	MAT	−2.03	0.043	267	265	97.28	89.144
	AP	−6.02	0.000				
Wood resin	MAT	2.48	0.014	267	265	51.766	48.595
	AP	3.96	0.000				
Needle phenolics	MAT	−3.73	0.000	267	265	98.674	88.972
	AP	−5.38	0.000				
Wood phenolics	MAT	−1.05	0.295	267	265	49.618	44.821
	AP	−5.26	0.000				

**Figure 4.**
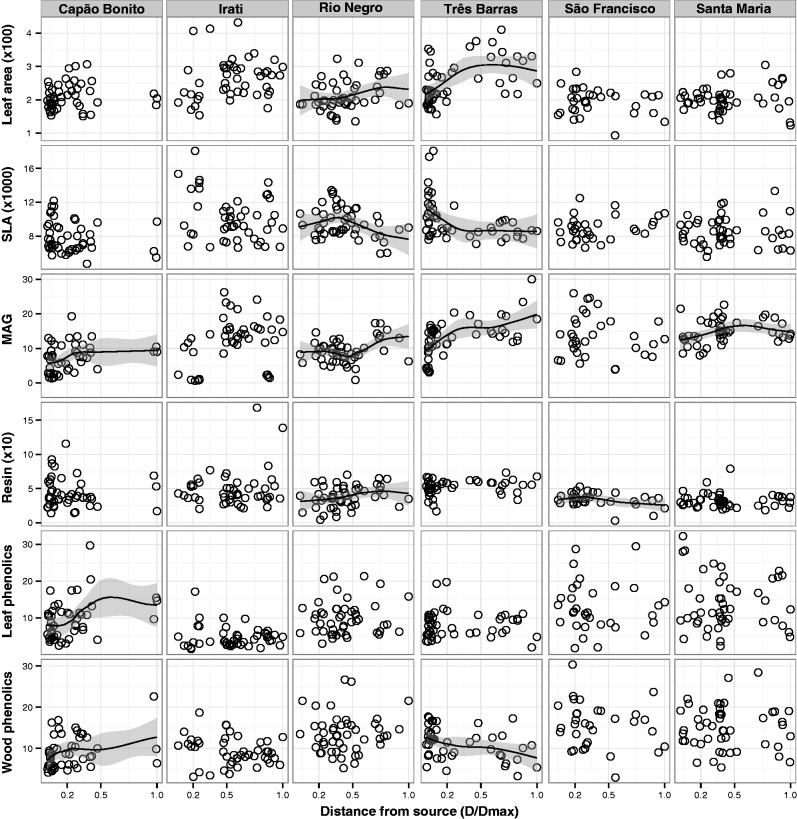
Relationship between productivity, growth, and defence traits and dispersal distance (*D*/*D*_max_, see Methods section for details). Scatterplots of leaf area (mm^2^), specific leaf area (SLA, mm^2^/g), mean annual growth (MAG, mm/year), non-volatile wood resin content (mg/g) and total needle phenolic content (mg/g) across the invasion gradient (normalized spread distance) for each invasive population. Lines represent local polynomial regression fit and grey shades are the 95% confidence intervals.

Not only were there between-population variations but also the invasive populations were rapidly changing along each invasive range expansion (Fig. 4 and Table 3). We tested rapid evolution along the six invasive range expansions using linear mixed-effect models with distance from plantation and provenance as fixed effects and plant age as random effect. Two populations (Rio Negro and Três Barras) showed increases in LAs (Fig. 4 and Table 3, pseudo-*r*^2^ = 0.08 and 0.26, *P* = 0.045 and *P* < 0.001, respectively) and decreases in SLA (Fig. 4 and Table 3, pseudo-*r*^2^ = 0.1 and 0.2, *P* = 0.02 and 0.005, respectively) during range expansion. Four populations (Capão Bonito, Rio Negro, Três Barras and Santa Maria) showed faster growth rates at the leading edge of the invasion front in comparison with plants at the rear edge (Fig. 4 and Table 3, pseudo-*r*^2^ = 0.1, 0.1, 0.3, 0.1, and *P* = 0.03, 0.03, <0.001, 0.02, respectively). In two of these cases (Irati and Três Barras), MAG increased 2-fold in 40 years (Fig. 4). None of the populations showed decreases in MAG. In terms of constitutive and inductive plant defences, non-volatile resin content increased in plants along one invasion gradient (Rio Negro, pseudo-*r*^2^ = 0.1, *P* = 0.03) and decreased in a second (São Francisco, pseudo-*r*^2^ = 0.1, *P* = 0.04), total needle phenolic content increased in plants along one invasion gradient (Capão Bonito, pseudo-*r*^2^ = 0.1, *P* = 0.02) and total wood phenolic content increased in plants along the Capão Bonito invasion gradient (pseudo-*r*^2^ = 0.1, *P* = 0.03) and decreased in a second (Três Barras, pseudo-*r*^2^ = 0.1, *P* = 0.04).

**Table 3.** Summary results of likelihood ratio test among linear mixed-effect models. Pseudo-*r*^2^ represents the variance explained by fixed effects. Degrees of freedom (d*f*) are reported for the likelihood ratio tests. SLA, specific leaf area; MAG, mean annual growth.

		Capão Bonito			Irati			Rio Negro			Três Barras			São Francisco de Paula			Santa Maria		
		d*f*	Pseudo-*r*^2^	*P*	d*f*	Pseudo-*r*^2^	*P*	d*f*	Pseudo-*r*^2^	*P*	d*f*	Pseudo-*r*2	*P*	d*f*	Pseudo-*r*^2^	*P*	d*f*	Pseudo-*r*^2^	*P*
Leaf area	Full	5	0.012	0.551	5	0.017	0.811	5	0.179	0.016	5	0.283	< 0.001	5	0.050	0.198	5	0.024	0.447
	Invasion	4	0.006	0.615	4	0.003	0.702	4	0.080	0.045	4	0.262	< 0.001	4	0.049	0.196	4	0.016	0.368
	Provenance	4	0.003	1.000	4	0.016	< 0.001	4	0.074	1.000	4	0.005	1.000	4	0.002	1.000	4	0.012	1.000
SLA	Full	5	0.084	0.108	5	0.110	0.268	5	0.103	0.024	5	0.303	0.003	5	0.056	0.232	5	0.004	0.965
	Invasion	4	0.040	0.169	4	0.030	0.184	4	0.103	0.023	4	0.165	0.005	4	0.039	0.235	4	0.000	0.969
	Provenance	4	0.032	1.000	4	0.088	< 0.001	4	0.002	1.000	4	0.136	1.000	4	0.017	1.000	4	0.004	< 0.001
MAG	Full	5	0.191	0.010	5	0.158	0.113	5	0.098	0.032	5	0.338	< 0.001	5	0.049	0.205	5	0.092	0.026
	Invasion	4	0.096	0.032	4	0.082	0.070	4	0.098	0.030	4	0.335	< 0.001	4	0.049	0.199	4	0.092	0.017
	Provenance	4	0.068	1.000	4	0.093	< 0.001	4	0.002	1.000	4	0.002	1.000	4	0.002	1.000	4	0.016	1.000
Wood resin	Full	5	0.020	0.473	5	0.034	0.211	5	0.135	0.014	5	0.117	0.179	5	0.146	0.037	5	0.004	0.971
	Invasion	4	0.007	0.557	4	0.034	0.203	4	0.100	0.025	4	0.041	0.166	4	0.114	0.040	4	0.000	0.896
	Provenance	4	0.009	< 0.001	4	0.001	1.000	4	0.020	1.000	4	0.081	< 0.001	4	0.033	1.000	4	0.004	< 0.001
Leaf phenolics	Full	5	0.110	0.021	5	0.021	0.527	5	0.008	0.549	5	0.001	0.850	5	0.034	0.760	5	0.036	0.694
	Invasion	4	0.109	0.021	4	0.007	0.581	4	0.007	0.562	4	0.001	0.844	4	0.002	0.770	4	0.008	0.521
	Provenance	4	0.000	1.000	4	0.015	< 0.001	4	0.000	1.000	4	0.000	1.000	4	0.032	< 0.001	4	0.033	< 0.001
Wood phenolics	Full	5	0.116	0.048	5	0.041	0.243	5	0.010	0.663	5	0.147	0.012	5	0.023	0.468	5	0.005	0.616
	Invasion	4	0.094	0.034	4	0.040	0.257	4	0.005	0.634	4	0.131	0.012	4	0.019	0.453	4	0.005	0.615
	Provenance	4	0.036	1.000	4	0.005	1.000	4	0.006	< 0.001	4	0.019	1.000	4	0.008	1.000	4	0.000	1.000

Some instances revealed that evolutionary forces did not produce phenotypic changes along the invasive range expansions for the traits measured. Some trait variations were explained by the genetic provenance of the plants (i.e. genetically conserved) (Table 3). In Irati, LA (pseudo-*r*^2^ = 0.02, *P* < 0.001), SLA (pseudo-*r*^2^ = 0.09, *P* < 0.001), MAG (pseudo-*r*^2^ = 0.09, *P* < 0.001) and total phenolic (pseudo-*r*^2^ = 0.02, *P* < 0.001) values were explained by the plants genetic provenance. Ancestry also explained SLA in Santa Maria (pseudo-*r*^2^ = 0.004, *P* < 0.001), wood resin in Capão Bonito, Três Barras and Santa Maria (pseudo-*r*^2^ = 0.009, pseudo-*r*^2^ = 0.08, pseudo-*r*^2^ = 0.004, all *P* < 0.001), needle total phenolic content in São Francisco and Santa Maria (pseudo-*r*^2^ = 0.02 and 0.03, and *P* < 0.001 for both) and wood total phenolic content in Rio Negro (pseudo-*r*^2^ = 0.006, *P* < 0.001).

**Figure 5.**
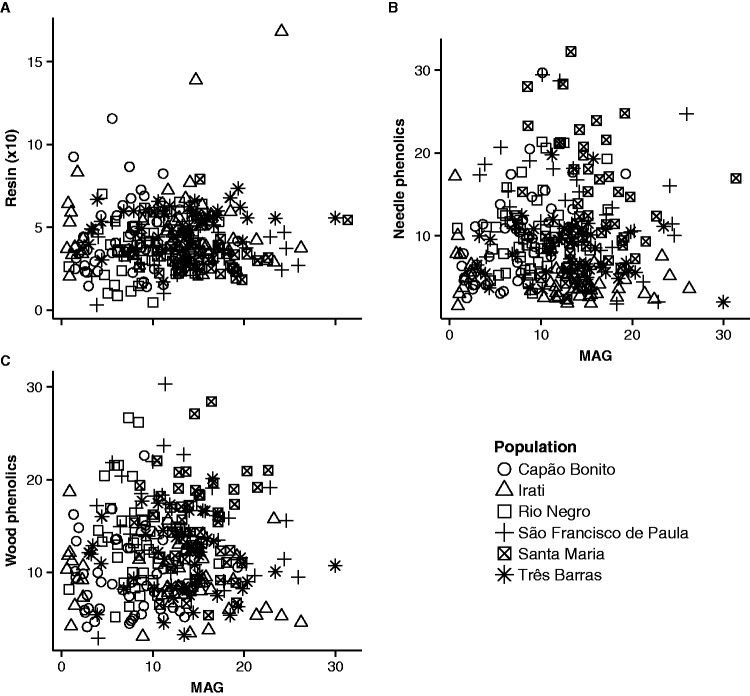
Relationship between chemical defences and mean annual growth in plants across the six studied naturalized populations. (A) Relationships between resin contents and mean annual growth, (B) relationships between needle total phenolic contents and mean annual growth and (C) relationships between wood total phenolic contents and mean annual growth. Dots of different shapes represent different naturalized populations. None but one of the relationships were statistically significant (needle phenolics and MAG in Capão Bonito; see text for details).

Our analyses of potential trade-offs between growth and chemical defences yielded no significant relationships (*P* > 0.05) among MAG and wood resin, wood total phenolics, or needle total phenolics in all but two models (Fig. 5). In Irati, concentrations of total phenolics in needles were lower in plants with higher MAG (*r*^2^ = 0.12, *P* = 0.02), but a single outlier (IRT6) with very high phenolic content drove this result. Removing the outlier from the model yielded a non-significant relationship (*P* = 0.09). In Capão Bonito, contrary to all expectations, concentrations of total phenolics in needles and MAG were positively associated (*r*^2^ = 0.22, *P* < 0.001).

**Figure 6.**
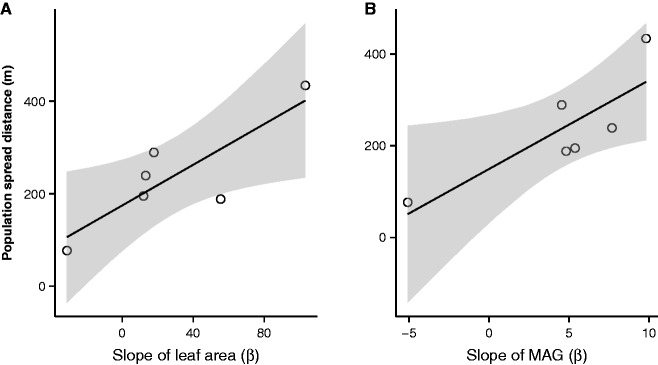
Relationship between evolution in leaf area and growth and invasive range expansion. Regression line and 95% confidence intervals (grey shade) of the relationship between the rate of evolution in (A) leaf area and (B) mean annual growth (MAG) for the six invasive populations. Evolution is represented by the mean slope of the mixed models between normalized distance of spread and trait value (Fig. 4).

When testing how rate of evolutionary responses affected invasive ranges expansions, we found that rate of change of increased MAG and rate of change of increased LA were positively associated to the total spread distances of the invasive populations (*r*^2^ = 0.7 and *P* = 0.04 for both models, Fig. 6). However, although plant MAG and LA were not correlated (*P* = 0.07) and both were positively related to total spread distance, the full model was not significant (*P* = 0.2). This was probably due to the small sample size (*n* = 6 populations).

In a genome-wide association analysis, we detected one gene associated with LA (SNP_210094-Pita) and two genes associated with MAG (SNP_215552-Pita and SNP_225711-Pita). SNP_210094-Pita is related to phosphorus assimilation by the roots (GenBank Acc: BE496394) and phosphorous has been experimentally shown to affect LA in forest trees (Herbert and Fownes 1995). SNP_215552-Pita is related to root development (GenBank Acc: CO198215), drought resistance (GenBank Acc: CF392949) and fungal resistance (GenBank Acc: DR093744). SNP_225711-Pita is associated with wood formation (GenBank Acc: BE496394).

## Discussion

Our results show strong evidences for rapid phenotypic change in all six invasive populations, providing support for our first hypothesis. However, contrary to our second hypothesis, patterns of phenotypic changes did not match patterns of genetic change across populations, and each population showed a unique pattern of phenotypic change along the invasion gradient. Surprisingly, also contrary to our initial expectations, populations did not exhibit trade-offs between evolution in growth and chemical defences. In one instance (Capão Bonito), there was a positive association between MAG and leaf phenolic content. Finally, supporting our last hypothesis, we found positive associations between rates of rapid change in plant growth (MAG) and productivity (LA) and the rates of population spread. The mismatches observed between phenotypic changes, genetic makeup of the plants and climate patterns suggest part of the variation found may be caused by phenotypic plasticity or genetic drift. Although populations are changing genetically along the invasion gradients (Zenni *et al.* 2014), the traits measured might be responding to the environment without a underlying genetic change. Also, the genetic and phenotypic changes might not be the result of selection, but the result of genetic drift and founder effects (van Kleunen and Fischer 2008; Monty *et al.* 2013).

A previous study in this system showed that climate was one of the factors determining the patterns of genetic change across the six populations (Zenni *et al.* 2014). Accordingly, climate (mean annual temperature and annual precipitation) also partially explained patterns of variation in LA, SLA, MAG, wood resin and leaf and wood phenolic contents among populations (Fig. 3). The strongest association was between MAG and annual precipitation, where growth rates were higher in wetter locations. Although statistically significant, mean annual temperature did not strongly affect the growth rates of plants. Relationships between *P. taeda* phenotypic patterns and climatic factors had also been reported in the species native range (e.g. Eckert *et al.* 2010). For other systems, climate has also been shown to be an important factor effecting population growth, spread, or retraction (Chen *et al.* 2011; Pfeifer-Meister *et al.* 2013; Kerr *et al.* 2015). In our study, the fact that all populations started from replicated introduction pools, and genotypic and phenotypic responses to each climate were observed during range expansions in less than 40 years, suggests climate may be an important driver of evolution for invasive populations. Under new climates, either owing to human-meditated introductions to a new range or to climate change, invasive populations may be the ones able to adapt quickly to the new environment.

Climate not only can drive ecological patterns of plant chemical defences but also the evolution of constitutive chemical defences in pines may be driven by it (Moreira *et al.* 2014). Our results seem to support this notion (Fig. 3), although we measured total phenolic content, which includes both constitutive and inducible defences. In the current study, we found differences in chemical defence traits both among and within populations (Table 2 and Figs 2 and 4). The among population differences may be due to ecological responses of populations to each environment (climate and habitat), whereas the changes along each invasion gradient may be showing evolutionary responses of each population to each environment. Considering that the among population differences are stronger than the within population changes, it is not possible to determine if adaptive evolution is occurring in chemical defences in the six studied populations or if differences are due to genetic drift caused by founder effects (Monty *et al.* 2013). However, it is possible evolution in the studied defence traits is occurring, but the effects were not detected. Another study, disentangling constitutive and inducible defences would be necessary to address the genotypic (constitutive), phenotypic (constitutive and inducible) or epigenetic components of defence responses.

The production of chemical defences is costly, and increased resource allocation to defences tends to result in disproportionally high decreases in growth rates for plants (Mithöfer and Boland 2012). Consequently, if non-native plants escape from natural enemies during introduction and are able to afford neglecting their chemical defences to favour growth, reproduction, and dispersal traits, they may become invasive (Callaway and Ridenour 2004; Felker-Quinn *et al.* 2013). However, in our study, we generally found no relationship between investments in plant growth, productivity and chemical defences. Plants showed increased or kept existing resource use strategies, high growth rates and levels of chemical defences. The quantities of resin and total phenolics we found were similar to quantities reported for the species by other studies in other regions (Moreira *et al.* 2014), indicating that *P. taeda* plants in the six studied populations had levels of chemical defences similar to the species' average. Although investments in chemical defences varied across populations, we did not find a consistent trend towards increased or decreased investments along the invasion gradients as we did for MAG. On one hand, resin and phenolics total contents might be a plastic response of populations and, given the lack of important natural enemies in the introduced ranges, there are no selection pressures for increased chemical defences. Or populations show a slower pace of change for chemical defences than for growth rates given the complexity of the metabolic processes involved in producing chemical defences (Mithöfer and Boland 2012). On the other hand, there may be no limiting resources requiring a trade-off between chemical defences and growth and increase growth rates are simply a result of favourable climatic conditions for *P. taeda* (e.g. Nuñez and Medley 2011).

Although the invasion literature advocates that high SLAs and fast growth have positive associations with woody plant invasiveness (Grotkopp and Rejmánek 2007; Zenni and Simberloff 2013), it has also been suggested that natural selection can reduce a population invasive potential (Lankau *et al.* 2009). Our data suggest that rapid evolution, either via natural selection or genetic drift, can result in populations with increased invasive potential in comparison to the original introduction pool. In most of our cases, plants at the leading edges of the invasion fronts grew faster and were more productive, on an average, than plants at the rear edges (Fig. 4). These effects were mediated by climate, suggesting that in conditions adverse for the species, evolutionary changes may not be so pronounced. However, in none of the cases did populations showed slower growth rates or lower productivities at the leading edges. We found two populations (Rio Negro and Três Barras) that produced lower SLA along the invasion gradients, besides being actively spreading and invading. Four populations increased growth rates along the invasion gradients. Although our results seem to contradict the established literature (Tecco *et al.* 2010; Porté *et al.* 2011), we believe that the decrease in SLA combined with increases in LA may be explained as a response of *P. taeda* plants to the forest ecosystems of Rio Negro and Três Barras. MAG results were in agreement with the established literature that fast growth is a key invasion trait (Lamarque *et al.* 2011).

Also supporting the idea that rapid evolutionary change in growth rates and productivity can increase the invasive potential of populations (Lamarque *et al.* 2011; Monty *et al.* 2013; Sargent and Lodge 2014), we found strong relationships between MAG and LA and total spread of populations. Populations with greater rate of change towards increased growth rates and LA spread farther in the 40 years period encompassed by this study (Fig. 6). However, it is also possible that greater spread could lead to higher growth if plants are experiencing less competition at the leading edge of the invasion front, or if growth and spread were both correlated to a third factor. Nevertheless, the strong effect rapid evolution can have on populations’ invasiveness suggests that invasions must be dealt with sooner rather than latter.

Several of the statistical models including only genetic provenance as a factor were significantly different than the null models, but overall they had low explanatory power (Table 2). It is possible that genetic provenance is a coarse scale to test fine evolutionary changes in populations. The fine-scaled genome-wide association analyses, where trait values were tested for each one of the 94 genes included in this study, we found three genes associated with the traits measured. Given that we used putatively functional markers, we expected that more of the genes would be associated with traits. A possible explanation for the few significant associations found is that most traits result from the expression of many genes, instead of only a few genes of large effect. Also, because we measured plants growing in natural environments and not in a controlled homogenous setting (e.g. greenhouse), phenotypic plasticity may be masking the role of specific genes in trait expression. Taken together with previous findings on the study system that several genes were undergoing rapid changes in genotype frequencies (Zenni and Hoban 2015b), the results support our claim that evolution is occurring in this system and changes were not caused only by phenotypic plasticity.

Because our study took place in a natural setting, and not a controlled environment, it is subject to several caveats. It is possible some of the variations in traits values could be explained by the age and size of the trees, as well as by micro-environmental factors that differ for each individual tree, and that may vary along each of the invasion gradients. We tried to address the effect of tree size and age in the statistical models by having tree age as a random effect in the mixed models and by using only the variance explained by the fixed effects in subsequent analyses and interpretations of the model results. We were unable to disentangle the relative importance of adaptive evolution, genetic drift and phenotypic plasticity for the traits measured. However, given the structure of our statistical models, it is likely that plastic responses only decreased the chances of us finding significant relationships between trait evolution and invasion spread. We still found a number of significant associations. Further, although we acknowledge the existence and the potential importance of micro-environmental factors in affecting trait values on each plant (adaptive and plastic), we think they make our findings even more remarkable. We showed that despite all potential micro-environmental variations in each of six naturalized population spanning an 840 km transect, four climate zones, and four decades of natural processes, rapid evolution in several functional traits could still be detected along invasion gradients.

## Conclusions

In summary, our study provides strong support for the role of rapid evolution on the success of non-native range expansions of an invasive species. We also show that evolutionary changes are in part associated with climate, but are mostly fine-scaled and context-specific, even though invasive populations started from fully replicated introduction events. The results of this study highlight the unique nature of the ecological and evolutionary dynamics of each population, suggesting that predicting invasion trajectories may be daunting. Further, we demonstrate that the potential for rapid evolution can affect the capacity of populations to cope with new environments. Taken together, the capacity for adaptation to different conditions, evolution of increased growth rates, and the lack of trade-offs between growth and defences make *P. taeda* an invasive species that requires management and control before its spread reach large areas.

## Sources of Funding

Our work was funded by CNPq-Brazil (313926/2014-0).

## Contributions by the Authors

R.D.Z. conceived the study, collected data, measured resin contents, did statistical analyses and wrote the manuscript. W.L.C. collected data, measured SLAs and mean annual growths. G.S. measured phenolic contents. All authors discussed the results and commented on the manuscript.

## Conflict of Interest Statement

None declared.

## Supplementary Material

Supplementary Data

## References

[plw048-B1] Berthouly-Salazar C Hui C Blackburn TM Gaboriaud C Rensburg BJ Vuuren BJ Roux JJ. 2013 Long‐distance dispersal maximizes evolutionary potential during rapid geographic range expansion. Molecular Ecology 22:5793–5804. 24192018 10.1111/mec.12538

[plw048-B2] Blackburn TM Pyšek P Bacher S Carlton JT Duncan RP Jarošík V Wilson JRU Richardson DM. 2011 A proposed unified framework for biological invasions. Trends in Ecology & Evolution 26:333–339. 21601306 10.1016/j.tree.2011.03.023

[plw048-B3] Blossey B Nötzold R. 1995 Evolution of increased competitive ability in invasive nonindigenous plants: a hypothesis. Journal of Ecology 83:887–889.

[plw048-B4] Burton OJ Phillips BL Travis JMJ. 2010 Trade-offs and the evolution of life-histories during range expansion. Ecology Letters 13:1210–1220. 20718846 10.1111/j.1461-0248.2010.01505.x

[plw048-B5] Buswell JM Moles AT Hartley S. 2011 Is rapid evolution common in introduced plant species? Journal of Ecology 99:214–224.

[plw048-B6] Callaway RM Ridenour WM. 2004 Novel weapons: invasive success and the evolution of increased competitive ability. Frontiers in Ecology and the Environment 2:436–443.

[plw048-B7] Caplat P Cheptou PO Diez J Guisan A Larson BMH Macdougall AS Peltzer DA Richardson DM Shea K van Kleunen M Zhang R Buckley YM. 2013 Movement, impacts and management of plant distributions in response to climate change: insights from invasions. Oikos 122:1265–1274.

[plw048-B8] Chen I-C Hill JK Ohlemüller R Roy DB Thomas CD. 2011 Rapid range shifts of species associated with high levels of climate warming. Science 333:1024–1026. 21852500 10.1126/science.1206432

[plw048-B9] Colautti RI Barrett SCH. 2013 Rapid adaptation to climate facilitates range expansion of an invasive plant. Science 342:364–366. 24136968 10.1126/science.1242121

[plw048-B10] Coley PD Bryant JP Chapin FS. 1985 Resource availability and plant antiherbivore defense. Science 230:895–899. 17739203 10.1126/science.230.4728.895

[plw048-B11] Colomer-Ventura F Martínez-Vilalta J Zuccarini P Escolà A Armengot L Castells E. 2015 Contemporary evolution of an invasive plant is associated with climate but not with herbivory. Functional Ecology. 29:1475–1485.

[plw048-B12] Donaldson JE Hui C Richardson DM Robertson MP Webber BL Wilson JR. 2014 Invasion trajectory of alien trees: the role of introduction pathway and planting history. Global Change Biology 20:1527–1537. 24343918 10.1111/gcb.12486

[plw048-B13] Eckert AJ van Heerwaarden J Wegrzyn JL Nelson CD Ross-Ibarra J González-Martínez SC Neale DB. 2010 Patterns of population structure and environmental associations to aridity across the range of loblolly pine (*Pinus taeda* L., Pinaceae). Genetics 185:969–982. 20439779 10.1534/genetics.110.115543 PMC2907212

[plw048-B14] Felker-Quinn E Schweitzer JA Bailey JK. 2013 Meta-analysis reveals evolution in invasive plant species but little support for Evolution of Increased Competitive Ability (EICA). Ecology and Evolution 3:739–751. 23531703 10.1002/ece3.488 PMC3605860

[plw048-B15] Forsman A Wennersten L. 2015 Inter-individual variation promotes ecological success of populations and species: evidence from experimental and comparative studies. Ecography. doi:10.1111/ecog.01357:n/a-n/a.

[plw048-B16] Forsman A Wennersten L Karlsson M Caesar S. 2012 Variation in founder groups promotes establishment success in the wild. Proceedings of the Royal Society B: Biological Sciences. doi:10.1098/rspb.2012.0174. 10.1098/rspb.2012.0174 PMC3367781 22456885

[plw048-B17] Grotkopp E Rejmánek M. 2007 High seedling relative growth rate and specific leaf area are traits of invasive species: phylogenetically independent contrasts of woody angiosperms. American Journal of Botany 94:526–532. 21636422 10.3732/ajb.94.4.526

[plw048-B18] Gurevitch J Fox GA Wardle GM Inderjit TD. 2011 Emergent insights from the synthesis of conceptual frameworks for biological invasions. Ecology Letters 14:407–418. 21513009 10.1111/j.1461-0248.2011.01594.x

[plw048-B19] Herbert DA Fownes JH. 1995 Phosphorus limitation of forest leaf area and net primary production on a highly weathered soil. Biogeochemistry 29:223–235.

[plw048-B20] Hijmans RJ Cameron SE Parra JL Jones PG Jarvis A. 2005 Very high resolution interpolated climate surfaces for global land areas. International Journal of Climatology 25:1965–1978.

[plw048-B21] Keane RM Crawley MJ. 2002 Exotic plant invasions and the enemy release hypothesis. Trends in Ecology & Evolution 17:164–170.

[plw048-B22] Kerr JT Pindar A Galpern P Packer L Potts SG Roberts SM Rasmont P Schweiger O Colla SR Richardson LL Wagner DL Gall LF Sikes DS Pantoja A. 2015 Climate change impacts on bumblebees converge across continents. Science 349:177–180. 26160945 10.1126/science.aaa7031

[plw048-B23] Kuebbing S Simberloff D. 2015 Missing the bandwagon: nonnative species impacts still concern managers. NeoBiota 25:73–86.

[plw048-B24] Lamarque L Delzon S Lortie C. 2011 Tree invasions: a comparative test of the dominant hypotheses and functional traits. Biological Invasions 13:1969–1989.

[plw048-B25] Lankau RA Nuzzo V Spyreas G Davis AS. 2009 Evolutionary limits ameliorate the negative impact of an invasive plant. Proceedings of the National Academy of Sciences 106:15362–15367. 10.1073/pnas.0905446106 PMC2730356 19706431

[plw048-B26] Liu H Stiling P. 2006 Testing the enemy release hypothesis: a review and meta-analysis. Biological Invasions 8:1535–1545.

[plw048-B27] Mithöfer A Boland W. 2012 Plant defense against herbivores: chemical aspects. Annual Review of Plant Biology 63:431–450. 10.1146/annurev-arplant-042110-103854 22404468

[plw048-B28] Monty A Bizoux J-P Escarré J Mahy G. 2013 Rapid plant invasion in distinct climates involves different sources of phenotypic variation. PLoS One 8:e55627. 23383251 10.1371/journal.pone.0055627 PMC3559535

[plw048-B29] Moodley D Geerts S Richardson DM Wilson JRU. 2013 Different traits determine introduction, naturalization and invasion success in woody plants: Proteaceae as a test case. PLoS One 8:e75078. 24086442 10.1371/journal.pone.0075078 PMC3782508

[plw048-B30] Moran EV Alexander JM. 2014 Evolutionary responses to global change: lessons from invasive species. Ecology Letters 17:637–649. 24612028 10.1111/ele.12262

[plw048-B31] Moreira X Mooney KA Rasmann S Petry WK Carrillo-Gavilán A Zas R Sampedro L. 2014 Trade-offs between constitutive and induced defences drive geographical and climatic clines in pine chemical defences. Ecology Letters 17:537–546. 24818235 10.1111/ele.12253

[plw048-B32] Moreira X Zas R Sampedro L. 2012 Differential allocation of constitutive and induced chemical defenses in pine tree juveniles: a test of the optimal defense theory. PLoS One 7:e34006. 22470508 10.1371/journal.pone.0034006 PMC3314687

[plw048-B33] Nakagawa S Schielzeth H. 2013 A general and simple method for obtaining R2 from generalized linear mixed‐effects models. Methods in Ecology and Evolution 4:133–142.

[plw048-B34] Nuñez MA Medley KA. 2011 Pine invasions: climate predicts invasion success; something else predicts failure. Diversity and Distributions 17:703–713.

[plw048-B35] Parker IM Rodriguez J Loik ME. 2003 An evolutionary approach to understanding the biology of invasions: local adaptation and general-purpose genotypes in the weed Verbascum thapsus. Conservation Biology 17:59–72.

[plw048-B36] Pfeifer-Meister L Bridgham SD Little CJ Reynolds LL Goklany ME Johnson BR. 2013 Pushing the limit: experimental evidence of climate effects on plant range distributions. Ecology 94:2131–2137. 24358697 10.1890/13-0284.1

[plw048-B37] Phillips BL Brown GP Webb JK Shine R. 2006 Invasion and the evolution of speed in toads. Nature 439:803–803. 16482148 10.1038/439803a

[plw048-B38] Porté AJ Lamarque LJ Lortie CJ Michalet R Delzon S. 2011 Invasive Acer negundo outperforms native species in non-limiting resource environments due to its higher phenotypic plasticity. BMC Ecology 11:28. 22115342 10.1186/1472-6785-11-28 PMC3275484

[plw048-B39] Prentis PJ Wilson JRU Dormontt EE Richardson DM Lowe AJ. 2008 Adaptive evolution in invasive species. Trends in Plant Science 13:288–294. 18467157 10.1016/j.tplants.2008.03.004

[plw048-B40] Pyšek P Jarošík V Hulme PE Pergl J Hejda M Schaffner U Vilà M. 2012 A global assessment of invasive plant impacts on resident species, communities and ecosystems: the interaction of impact measures, invading species' traits and environment. Global Change Biology 18:1725–1737.

[plw048-B41] Richardson DM Pyšek P. 2012 Naturalization of introduced plants: ecological drivers of biogeographical patterns. New Phytologist 196:383–396. 22943470 10.1111/j.1469-8137.2012.04292.x

[plw048-B42] Rius M Darling JA. 2014 How important is intraspecific genetic admixture to the success of colonising populations? Trends in Ecology & Evolution 29:233–242. 24636862 10.1016/j.tree.2014.02.003

[plw048-B43] Sargent LW Lodge DM. 2014 Evolution of invasive traits in nonindigenous species: increased survival and faster growth in invasive populations of rusty crayfish (*Orconectes rusticus*). Evolutionary Applications 7:949–961. 25469173 10.1111/eva.12198 PMC4211724

[plw048-B44] Schulte U Veith M Mingo V Modica C Hochkirch A. 2013 Strong genetic differentiation due to multiple founder events during a recent range expansion of an introduced wall lizard population. Biological Invasions 15:2639–2649.

[plw048-B45] Shimizu JY Higa AR. 1981 Variação racial do Pinus taeda L. no sul do Brasil até o sexto ano de idade. Boletim De Pesquisa Florestal 2:1–25.

[plw048-B46] Tecco PA Díaz S Cabido M Urcelay C. 2010 Functional traits of alien plants across contrasting climatic and land-use regimes: do aliens join the locals or try harder than them? Journal of Ecology 98:17–27.

[plw048-B47] van Kleunen M Fischer M. 2008 Adaptive rather than non-adaptive evolution of Mimulus guttatus in its invasive range. Basic and Applied Ecology 9:213–223.

[plw048-B48] Wardle DA Bardgett RD Callaway RM Van der Putten WH. 2011 Terrestrial ecosystem responses to species gains and losses. Science 332:1273–1277. 21659595 10.1126/science.1197479

[plw048-B49] Yelenik SG D'Antonio CM. 2013 Self-reinforcing impacts of plant invasions change over time. Nature 503:517–520. 24256723 10.1038/nature12798

[plw048-B50] Zenni RD. 2014 Analysis of introduction history of invasive plants in Brazil reveals patterns of association between biogeographical origin and reason for introduction. Austral Ecology 39:401–407.

[plw048-B51] Zenni RD Bailey JK Simberloff D. 2014 Rapid evolution and range expansion of an invasive plant are driven by provenance–environment interactions. Ecology Letters 17:727–735. 24703489 10.1111/ele.12278

[plw048-B52] Zenni RD Hoban SM. 2015a Data from: loci under selection during multiple range expansions of an invasive plant are mostly population-specific, but patterns are associated with climate. In: *Molecular ecology. Dryad digital repository: dryad digital repository.* 10.1111/mec.13234 25958932

[plw048-B53] Zenni RD Hoban SM. 2015b Loci under selection during multiple range expansions of an invasive plant are mostly population-specific, but patterns are associated with climate. Molecular Ecology 24:3360–3371. 25958932 10.1111/mec.13234

[plw048-B54] Zenni RD Simberloff D. 2013 Number of source populations as a potential driver of pine invasions in Brazil. Biological Invasions 15:1623–1639.

