# Supplementary material for: Rapid increase in growth and productivity can aid invasions by a non-native tree
Source: AoB Plants. 2016 Aug 2;8:plw048. doi: 10.1093/aobpla/plw048 (PMC4972472; doi:10.1093/aobpla/plw048)
Supplement: Supplementary Data [file supp_8_plw048_index.html]

Rapid increase in growth and productivity can aid invasions by a non-native tree — Supplementary Data 

# Rapid increase in growth and productivity can aid invasions by a non-native tree

## Supplementary Data

files

- Supplementary Data - docx file
