# Supplementary material for: Rapid increase in growth and productivity can aid invasions by a non-native tree
Source: AoB Plants. 2016 Aug 2;8:plw048. doi: 10.1093/aobpla/plw048 (PMC4972472; doi:10.1093/aobpla/plw048)
Supplement: Supplementary Data [file supp_plw048_aobplants-16016-s01.docx]

Table S1. Name and geographical coordinates of seed sources for the common garden experiments implemented in Brazil:

| **Seed source location ID** | **Seed source Location Name** | **Latitude (dec)** | **Longitude (dec)** | **Altitude (m)** |
| --- | --- | --- | --- | --- |
| 1 | Berkeley (SC) | 33.3 | -79.716667 | 8 |
| 2 | Marion (FL) | 29.1833333 | -82.5 | 24 |
| 3 | Worcester (MD) | 38.2 | -75.5 | 9 |
| 4 | Worcester (MD) 2 | 38.2 | -75.5 | 30 |
| 5 | Calcasieu (LA) | 30.3 | -93.3 | 15 |
| 6 | Livingston (LA) | 30.4 | -90.8 | na |
| 7 | Nacogdoches (TX) | 31.3 | -95 | 91 |
| 8 | Scott (MS) | 32.5 | -89.5 | 30 |
| 9 | Harrison (MS) | 30.5 | -89.1 | 46 |
| 10 | Stone (MS) | 30.6 | -89.1 | 107 |
| 11 | Jones (NC) | 35.2 | -77.8 | 9 |
| 12 | Oglethorp (GA) | 33.9 | -83.2 | 152 |
| 13 | Jones (GA) | 33 | -83.7 | 122 |
| 14 | Oglethorp (GA) 2 | 33.9 | -83.2 | 152 |
| 15 | Marion (FL) 2 | 29.25 | -82 | na |
| 16 | Scott (MS) 2 | 30.75 | -85.25 | 30 |
| 17 | Charleston (SC) | 30.75 | -90 | 8 |
| 18 | Forrest (MS) | 31 | -89.25 | 6 |
| 19 | Greene (GA) | 33.4 | -83.2 | 150 |
| 20 | Robeson (NC) | 34.9 | -79 | na |
| 21 | Durham (NC) | 36 | -79 | na |
| 22 | Chickasaw (MI) | 33.9 | -89 | na |
| 23 | Nottoway (VA) | 37.1 | -78 | 121 |
| 24 | Pasquotank (NC) | 36.3 | -76.2 | 5 |
| 25 | Worcester (MD) | 38.2 | -75.5 | 3 |
| 26 | Kent (MD) | 39.1 | -76.2 | 1 |
| 27 | Kershaw (MD) | 34.3 | -80.5 | 75 |
| 28 | Talladega (AL) | 33.3 | -86 | 250 |
| 29 | Angelina (TX) | 31 | -94.1 | 30 |
| 30 | Perquimans (NC) | 36.1666667 | -76.333333 | 7 |
| 31 | Lost pines (TX) | na | na | na |
| 32 | Florida | na | na | na |
